# Effects of elevated CO_2_ on phytoplankton during a mesocosm experiment in the southern eutrophicated coastal water of China

**DOI:** 10.1038/s41598-017-07195-8

**Published:** 2017-07-31

**Authors:** Xin Liu, Yan Li, Yaping Wu, Bangqin Huang, Minhan Dai, Feixue Fu, David A. Hutchins, Kunshan Gao

**Affiliations:** 1 State Key Laboratory of Marine Environmental Science, Xiamen University, 361005 Xiamen, Fujian China; 2 Department of Biological Sciences, University of Southern California, 3616 Trousdale Parkway, Los Angeles, California 90089 USA

## Abstract

There is a growing consensus that the ongoing increase in atmospheric CO_2_ level will lead to a variety of effects on marine phytoplankton and ecosystems. However, the effects of CO_2_ enrichment on eutrophic coastal waters are still unclear, as are the complex mechanisms coupled to the development of eutrophication. Here, we report the first mesocosm CO_2_ perturbation study in a eutrophic subtropical bay during summer by investigating the effect of rising CO_2_ on a model artificial community consisting of well-characterized cultured diatoms (*Phaeodactylum tricornutum* and *Thalassiosira weissflogii*) and prymnesiophytes (*Emiliania huxleyi* and *Gephyrocapsa oceanica*). These species were inoculated into triplicate 4 m^3^ enclosures with equivalent chlorophyll *a* (Chl-*a*) under present and higher partial pressures of atmospheric CO_2_ (*p*CO_2_ = 400 and 1000 ppmv). Diatom bloom events were observed in all enclosures, with enhanced organic carbon production and Chl-*a* concentrations under high CO_2_ treatments. Relative to the low CO_2_ treatments, the consumption of the dissolved inorganic nitrogen and uptake ratios of N/P and N/Si increased significantly during the bloom. These observed responses suggest more extensive and complex effects of higher CO_2_ concentrations on phytoplankton communities in coastal eutrophic environments.

## Introduction

At present, one of the most far-reaching global perturbations of the marine environment is caused by the massive invasion of fossil fuel CO_2_ into the ocean, making it the second largest sink for anthropogenic carbon dioxide after the atmosphere itself^1^. CO_2_ dissolved in seawater forms free H^+^ ions, lowering ocean pH and shifting dissolved inorganic carbon away from carbonate (CO_3_^2−^) towards more bicarbonate (HCO_3_^−^) and CO_2_. This global effect of anthropogenic CO_2_ emissions on ocean carbonate chemistry is of concern because it is already lowering the pH of the oceans, which may have ramifications for the growth, productivity and dominance of individual organisms or whole marine ecosystems^2^.

It has been suggested that the consequences of global ocean acidification will become more acute in the coastal zone, due to the decomposition of organic matter produced in eutrophic waters^3^. Coastal areas are complex and dynamic places in which environmental factors typically exhibit great spatial and temporal variability. For example, CO_2_ partial pressures (*p*CO_2_) in the inner estuary of the highly eutrophic Pearl River was found to range from 3380 to 4785 µatm in the summer, with a pH of 7.0–7.2^4^. Meanwhile, in a concurrent bloom in the outer estuary of the Pearl River *p*CO_2_ dropped rapidly to ~200 µatm, and pH rose to as high as 8.6^5^. This is mainly due to a variety of biogeochemical processes in coastal water, not from changes of CO_2_ in atmospheric concentrations. Terrigenous inputs, upwelling effects and biological activities (algal blooms, bacterial respiration) play important roles on the variations of *p*CO_2_ and pH in the water. The coastal acidification has been predicted to be over 10% faster compared to pelagic waters, as the decomposition of organic carbon by bacteria leads to an extra increase in CO_2_ concentration, usually associated with the processes of phytoplankton blooms and hypoxia in estuaries during summer^3^. Thus, although the partial pressure of CO_2_ in some stages of eutrophication is lower than the anthropogenically-influenced atmospheric *p*CO_2_, eutrophic coastal waters will still be under the influence of high *p*CO_2_ in the future^3^.

Efforts to understand potential consequences and feedbacks of increasing CO_2_ have been employed using both laboratory and mesocosm studies over scales ranging from genetic to ecosystem levels^6, 7^. Among the laboratory tests to investigate biological responses to ocean acidification, diazotrophic cyanobacteria, diatoms and prymnesiophytes (coccolithophores) are the most studied groups^8, 9^. In eutrophic coastal waters, diatoms and prymnesiophytes are dominant groups^8^, and responsible for a large fraction of oceanic primary production, playing an important role in marine ecosystems. Some typical species, such as *Phaeodactylum tricornutum* and *Emiliania huxleyi*, have been intensively studied with respect to their modes of C acquisition and various responses to changes in seawater CO_2_ at physiological, biochemical and molecular levels^2, 10^. However, since a majority of laboratory studies have investigated responses of single species, the knowledge obtained is difficult to extrapolate to these species’ responses to ocean acidification in natural complex environments. On the other hand, there are too many species in natural communities, including phytoplankton, zooplankton, and bacteria. In addition, natural communities in coastal waters have to confront to the complex influences of both natural change and human activities^2^. Therefore, we presently know little about how these organism responses scale up to the community and ecosystem levels and what the consequences are for marine food webs and biogeochemical cycles^6, 7, 11^. One way to bridge this gap between single species studies and highly complex natural communities is to test the mechanistic effects of global change factors on relatively less complex artificial communities composed of a few key phytoplankton groups^12^.

Mesocosm approaches are useful to shorten this gap between laboratory tests and *in-situ* investigations, and mixtures of well-studied phytoplankton species should be studied at mesocosm level to evaluate competition and succession under elevated CO_2_. Laboratory cultures are normally kept under stable conditions (e.g. constant light, temperature), while mesocosm enclosures are exposed to varying or fluctuating environmental factors, such as solar radiation and diel changes of temperature. Therefore, mesocosm enclosures are designed to approximate natural conditions in which environmental factors can be manipulated and closely monitored, and so provide a powerful tool to understand and forecast the effects of environmental changes on pelagic communities and the associated impacts on biogeochemical cycling^6^. In spite of this, recent findings show that there are still many unanswered questions using these approaches^7^. For example in mesocosm enclosures in the southern Norway, the inferred cumulative C:N stoichiometry of organic production increased with CO_2_ treatments at initial CO_2_ partial pressures of 350, 700 and 1,050 ppmv from 6.3 to 7.1 to 8.3 at the height of the bloom, respectively^9^. This suggests that ocean acidification may modify the stoichiometry of pelagic primary production which consumed up to 39% more dissolved inorganic carbon at increased *p*CO_2_ compared to the ambient level, whereas nutrient drawdown remained similar^1, 9^. However, considerable uncertainty about this finding was shown in other mesocosm tests (Table S1). During a CO_2_ perturbation study in Kongsfjorden on the west coast of Spitsbergen (Norway), carbon to nutrient uptake ratios were lower or higher than Redfield proportions during different phases of the experiment^13^. Another mesocosm study in the coastal waters of Korea also found the stoichiometry of elemental assimilation was insensitive to increasing *p*CO_2_ concentration and was close to the Redfield ratio of ∼6.6^14, 15^. In addition, effects of ocean acidification on phytoplankton uptake stoichiometry in coastal waters may result in a series of complicated changes in biogeochemical cycles^10^, such as altering the potential for phytoplankton growth limitation by nutrient elements such as P or Si over different spatial and temporal scales. Therefore, in highly productive coastal ecosystems, which support fisheries and other ecosystem services, unpredictable effects of ocean acidification ought to be intensively studied, since the biogeochemical response to increasing CO_2_ is obviously more complex than has been suggested from previous studies^7, 10, 13, 16^.

Under eutrophic conditions, nutrient limitation of phytoplankton growth could be alleviated and CO_2_ might be a limiting factor, especially during algal blooms. Subsequently, it is reasonable to hypothesize that growth of phytoplankton in eutrophic waters could be enhanced under elevated CO_2_ levels. In this study, we conducted a mesocosm CO_2_ perturbation study in a eutrophic subtropical bay in China during early summer, to investigate the effect of rising CO_2_ on a model phytoplankton community consisting of four well-studied phytoplankton isolates. These results are then used to discuss the following questions: i) What is the effect of high CO_2_ partial pressure on the growth of phytoplankton in eutrophic coastal waters? ii) How does elevated CO_2_ affect the competition between diatoms and prymnesiophytes (coccolithophores), the two most studied taxonomic groups in response to ocean acidification? and iii) Will the effects of high *p*CO_2_ on phytoplankton uptake stoichiometry in coastal waters alter the potential for limitation by nutrients such as N, P, or Si?

## Results

### Temporal evolution of the carbon dioxide system

**Figure 1:**
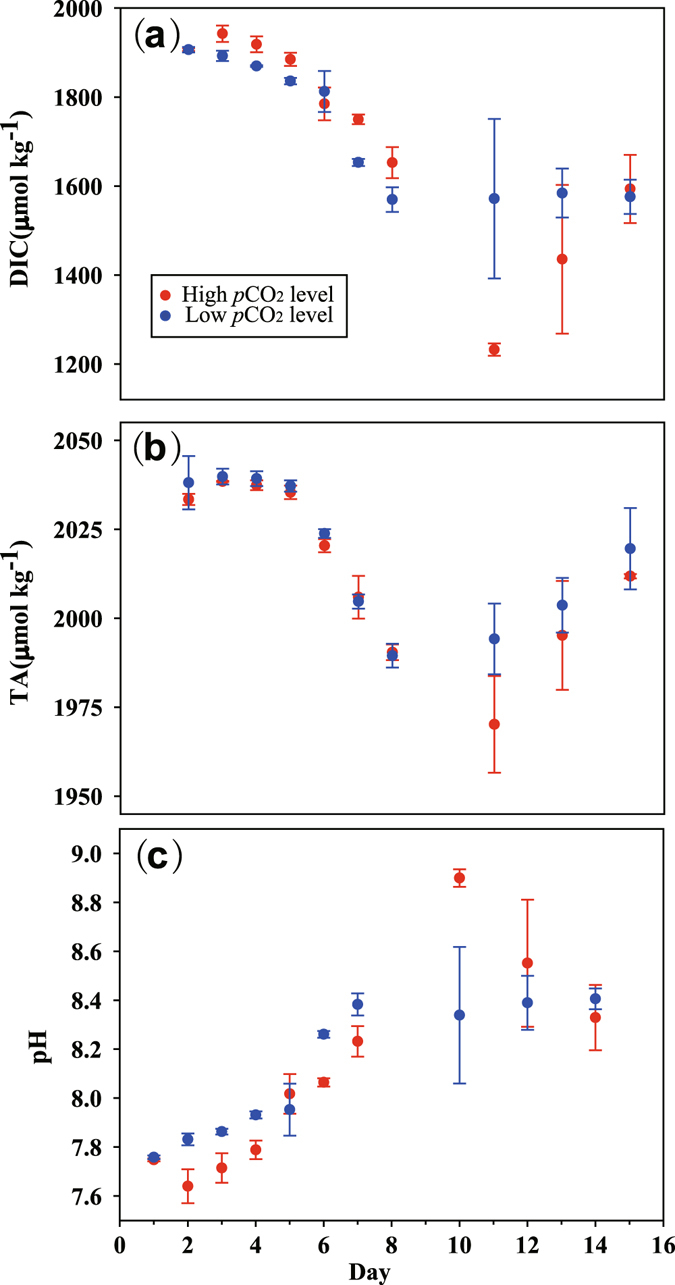
Temporal variations of concentrations of dissolved inorganic carbon (DIC, panel a), total alkalinity (TA, **b**) and pH (**c**) in 6 enclosures perturbed by bubbling with ambient air (400 ppmv CO_2_, Low CO_2_ level) or an air/CO_2_ mixture at a concentration of 1000 ppmv CO_2_ (High CO_2_ level) over a two week incubation. Symbols are the means and error bars are the standard errors of three replicate enclosures.

**Figure 2:**
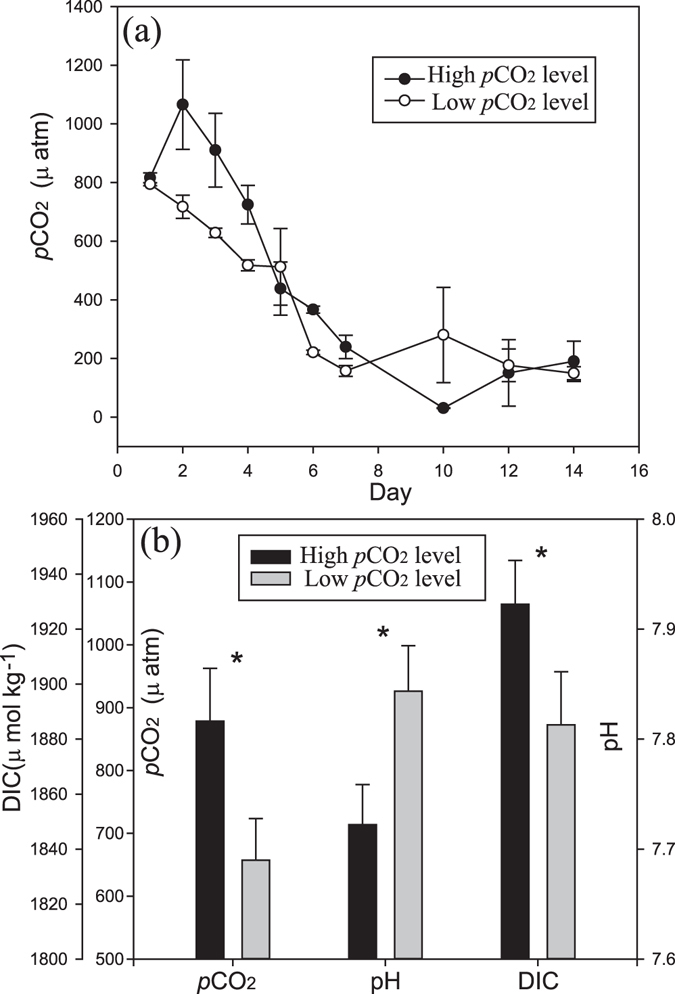
Temporal variations of the CO_2_ partial pressure in the seawater (*p*CO_2_, μatm, panel a) in 6 enclosures perturbed by bubbling with ambient air (400 ppmv CO_2_, Low CO_2_ level) or an air/CO_2_ mixture at a concentration of 1000 ppmv CO_2_ (High CO_2_ level) over a two week incubation, and comparisons on *p*CO_2_, pH and dissolved inorganic carbon (DIC) in the first 4 days (**b**). Symbols are the means and error bars are the standard errors of three replicate enclosures. Asterisk (*) indicate there are significantly differences between enclosures (p < 0.05).

The vertical variations on temperature and salinity in the enclosures were less than 0.1 °C and 0.1, respectively, indicating that the water was well mixed (Fig. S2). During the 15 days, there was a clear increase in temperature (mean value increase 2 °C) but only a slight increase in salinity (mean value increase 0.1). The initial dissolved inorganic carbon (DIC), total alkalinity (TA) and pH in the water were 2036 ± 9 (mean ± SD, the same below), 1907 ± 8 µmol kg^−1^ and 7.75 ± 0.01, respectively (Fig. 1). Consequently, the initial *p*CO_2_ in the water was 805 ± 22 µatm (Fig. 2a), indicating it was in fact already influenced by acidification, with a low pH and quite high DIC (Fig. 1). As expected, the initial concentrations of dissolved organic carbon (DOC) were quite high (day 1, 227 ± 15 µM).

Gradually with aeration from the CO_2_ enrichlors (400 and 1000 ppmv, HC and LC), there were significant differences between high and low CO_2_ treatments in pH, DIC and *p*CO_2_ in the water in the first 4 days (Fig. 2b, all p < 0.05). The mean *p*CO_2_ levels in the HC and LC treatments were 879.15 ± 145.76 and 658.05 ± 113.98 ppmv, with a significant differences of 221.10 ppmv in the first 4 days (Fig. 2b).

Along with the growth of phytoplankton and consequent depletion of nutrients, pH value increased in all enclosures (Fig. 1c). The *p*CO_2_ values dropped rapidly to ~200 µatm, and pH rose to over 8.5, indicating the impact of biological activity was first order. During the growth of phytoplankton, they were aerated continuously by different CO_2_ enrichments. Although lower pH values and higher *p*CO_2_ in the early phase were observed, quite high pH values (8.90 ± 0.06) and low *p*CO_2_ (31 ± 2 µatm) in the HC enclosures were observed on day 10 (Fig. 1).

### Temporal evolution of nutrients

**Figure 3:**
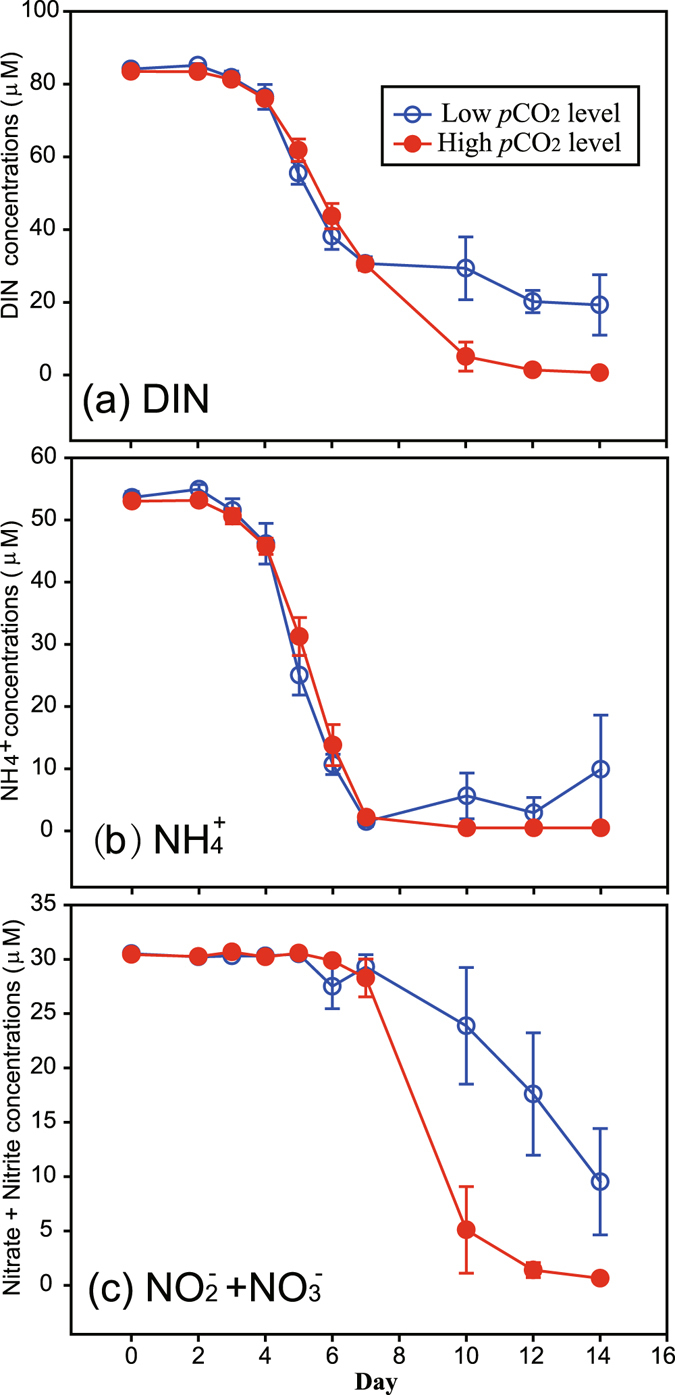
Temporal variations of concentrations of total dissolved inorganic nitrogen (DIN, panel a), NH_4_^+^ (**b**) and Nitrite + Nitrate (**c**) in 6 enclosures which were perturbed by bubbling with ambient air (400 ppmv CO_2_, Low CO_2_ level) or an air/CO_2_ mixture at a concentration of 1000 ppmv CO_2_ (High CO_2_ level). Symbols are the means and error bars are the standard errors of three replicate enclosures.

**Figure 4:**
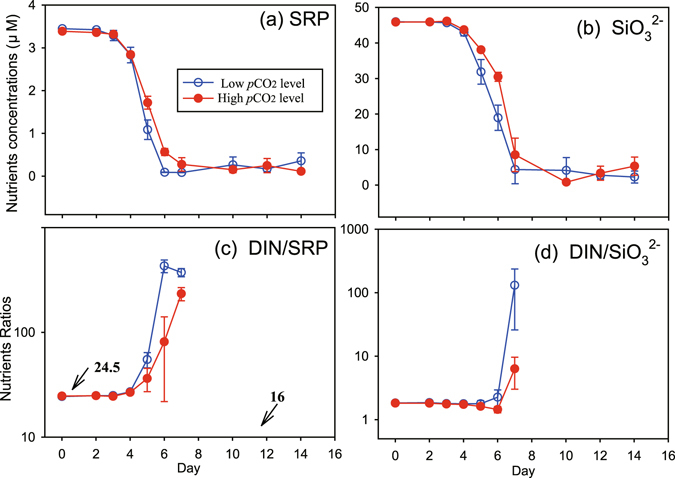
Temporal variations of concentrations of soluble reactive phosphorus (SRP, panel a), dissolved silicate, SiO_3_^2−^ (**b**), and ratios of dissolved inorganic nitrogen to soluble reactive phosphorus (**c**, DIN/SRP) and to SiO_3_^2−^ (**d**, DIN/SiO_3_^2−^) in 6 seawater enclosures which were perturbed by bubbling with ambient air (400 ppmv CO_2_, Low CO_2_ level) or an air/CO_2_ mixture at a concentration of 1000 ppmv CO_2_ (High CO_2_ level). Symbols are the means and error bars are the standard errors of three replicate enclosures. Nutrients ratio are shown during the nutrients replete period (day 0–7).

The initial filtered bay water used for the experiment was highly eutrophic, with nutrient concentrations in the initial waters of 30.48 ± 0.29, 53.32 ± 1.10 3.42 ± 0.05 and 45.92 ± 0.39 µM for NO_3_^−^ + NO_2_^−^ (NO_x_), NH_4_^+^, PO_4_^3−^ and SiO_3_^2−^, respectively (Figs 3 and 4). The composition of the dissolved inorganic nitrogen pool (DIN, NO_x_ + NH_4_^+^) indicates a very high proportion of NH_4_^+^ (Fig. 3). DIN concentrations dropped sharply after day 3 due to phytoplankton uptake, and the final drawdown in the HC treatment was significantly larger than that of the LC treatment (Fig. 3a, p < 0.01). Early in the nitrogen uptake process the preferential uptake of NH_4_^+^ is obvious, as the decrease of NO_x_ started only when NH_4_^+^ reached a minimum on day 7 (Fig. 3b). The DIN drawdown difference between treatments was due to the fact that the NO_x_ was not exhausted under the LC scenario (Fig. 3c).

**Figure 5:**
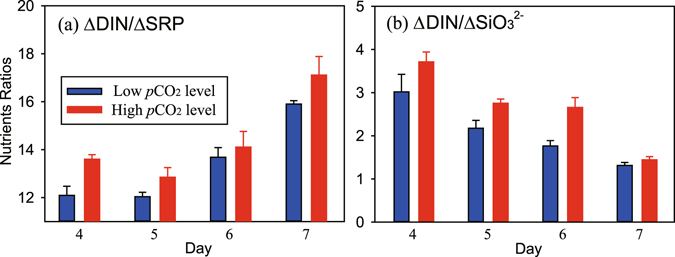
Temporal variations in nutrient consumption ratios of dissolved inorganic nitrogen to soluble reactive phosphorus (**a**, ΔDIN/ΔSRP) and to SiO_3_^2−^ (**b**, ΔDIN/Δ SiO_3_^2−^) during the nutrients replete period (day 4–7) in 6 seawater enclosures which were perturbed by bubbling with ambient air (400 ppmv CO_2_, Low CO_2_ level) or an air/CO_2_ mixture at a concentration of 1000 ppmv CO_2_ (High CO_2_ level). Columns are the means and error bars are the standard errors of three replicate enclosures.

The downward trends of SRP (soluble reactive phosphate) and SiO_3_^2−^ concentrations were consistent with that of ammonium, and the SRP concentrations were consumed completely first in LC treatments by day 6 (Fig. 4). Despite the eutrophic status of the collected experimental water, it was also potentially P-limited, as the SRP was first exhausted by day 6 and the initial DIN/SRP ratio was 24.5. This ratio exhibited a sustained increase along with the decline of inorganic nutrient concentrations in the seawater (Fig. 4c). Since more NOx was used in the HC enclosures, the DIN/SRP ratios in HC treatments after day 6 were significantly lower than those of the LC enclosures. Similar results were observed for variations in the DIN/SiO_3_^2−^ ratios (Fig. 4d). Ratios of net nutrient uptake (the difference with initial concentrations) also indicated treatment-dependent differences with significantly higher ratios of ΔDIN/ΔSRP and ΔDIN/ΔSiO_3_^2−^ in HC enclosures during the nutrient replete period (Fig. 5, day 4–7, n = 4, paired t-Test, p < 0.01). This suggests more consumption of DIN in the HC enclosures relative to SRP and SiO_3_^2−^.

### Variations on phytoplankton communities and carbon metabolism

**Figure 6:**
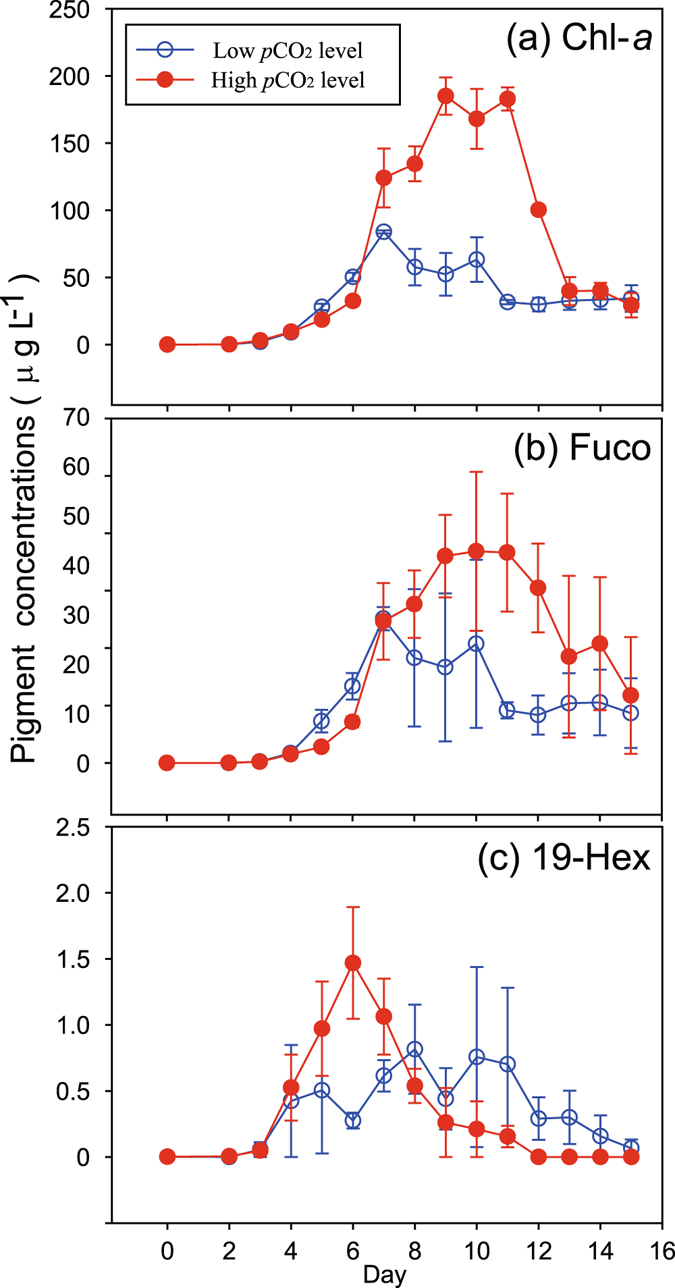
Temporal variations in concentrations of chlorophyll *a* (**a**) and diagnostic pigments of diatoms (**b**, Fucoxanthin) and prymnesiophytes (**c**, 19′hexanoyl-oxy-fucoxanthin, 19-Hex) in 6 seawater enclosures which were perturbed by bubbling with ambient air (400 ppmv CO_2_, Low CO_2_ level) or an air/CO_2_ mixture at a concentration of 1000 ppmv CO_2_ (High CO_2_ level). Symbols are the means and error bars are the standard errors of three replicate enclosures.

During the experiment, algal blooms were induced artificially in the enclosures (Fig. 6a). As there were no zooplankton grazers present, phytoplankton responses to the high CO_2_ treatments were strictly driven by “bottom-up” influences. Although the initial total chlorophyll *a* (Chl-*a*) concentrations of the four phytoplankton species were the same and the initial cell number of the prymnesiophytes was higher than that of the diatoms, the diatoms had an obvious advantage in the highly eutrophic water (Fig. 6). This is evident from the observation that the consumption of SiO_3_^2−^ was coupled to that of the other nutrients (Figs 3 and 4), as well as the increase in the diagnostic diatom pigment (Fucoxanthin, Fuco) (Fig. 6b).

**Figure 7:**
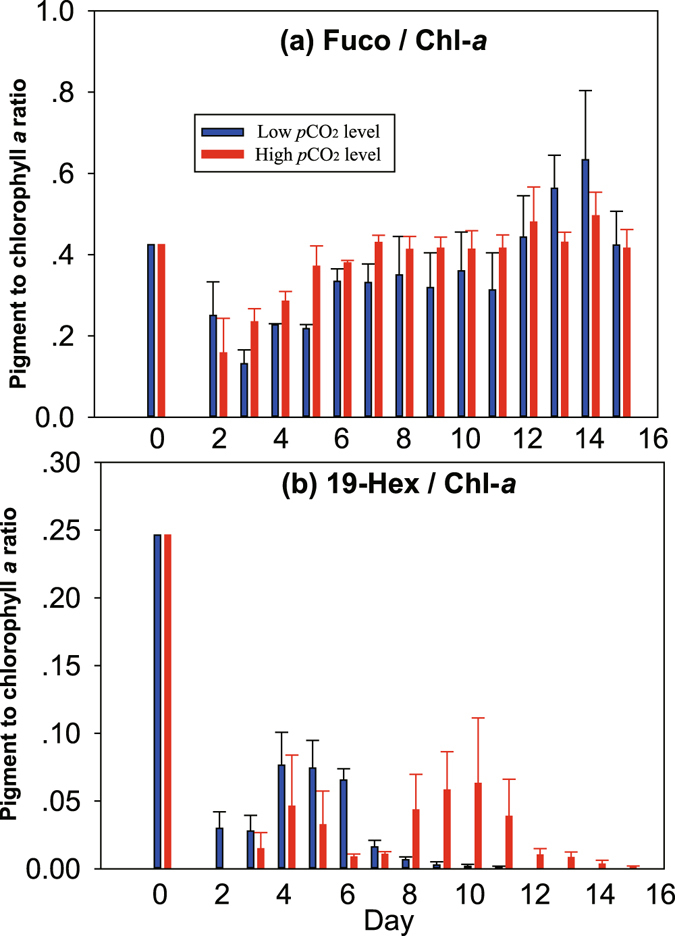
Temporal variations in ratios of fucoxanthin (**a**, Fuco) and 19′hexanoyl-oxy-fucoxanthin (**b**, Hex-Fuco) to chlorophyll *a* in 6 seawater enclosures which were perturbed by bubbling with ambient air (400 ppmv CO_2_, Low CO_2_ level) or an air/CO_2_ mixture at a concentration of 1000 ppmv CO_2_ (High CO_2_ level). Columns are the means and error bars are the standard errors of three replicate enclosures.

Phytoplankton Chl-*a* biomass reached a significantly higher concentration in the HC treatment on day 9 (185 ± 59 µg L^−1^), although its response seemed to show a short time lag (Fig. 6a). Prymnesiophytes rapidly responded in the HC enclosures as indicated by their diagnostic pigment 19′hexanoyl-oxy-fucoxanthin (19-Hex), reaching a peak on day 6 (1.4 ± 0.5 µg L^−1^, Fig. 6c). The temporal variations in the ratio of 19-Hex to Chl-*a* confirm that the contribution of prymnesiophytes to total Chl-*a* biomass was higher in the HC enclosures in the early phase (day 2–7), and the opposite was observed during days 8–14 (Fig. 7b). The ratios of Fuco to Chl-*a* exhibited no clear differences between the different treatments, both with a rising trend following a decline during the first few days (Fig. 7a). Based on the pigment ratios obtained in the mono-culture, the maximum Chl-*a* concentrations of diatom and prymnesiophytes in the HC enclosures were 166 ± 62 and 21 ± 33 µg L^−1^, while they were 76 ± 6 and 5.5 ± 3.5 µg L^−1^ in the LC treatments.

The production of CaCO_3_ (calcification) by prymnesiophytes in the HC enclosures (1.27 ± 0.07 µmol kg^−1^ d^−1^) was significantly lower than in the LC treatment (1.58 ± 0.12 µmol kg^−1^ d^−1^), while the production of particulate and dissolved organic carbon (POC and DOC) showed opposite trends (Table 1). These values in the HC enclosures (20.2 ± 7.6 and 29.7 ± 3.4 µM d^−1^, respectively) were significantly higher than those in the LC condition (8.8 ± 4.0 and 14.2 ± 4.8 µM d^−1^), respectively (p < 0.01). Thus, the ratios of particulate inorganic carbon (PIC) to POC in the HC enclosures were lower than those in the LC enclosures (Table 1).

**Table 1:** The overall particulate inorganic carbon (PIC), particulate organic carbon (POC) and dissolved organic carbon (DOC) production rates and PIC:POC ratios during the mesocosm incubations for 15 days under present and elevated partial pressure of CO_2_ (*p*CO_2_ = 400 and 1000 ppmv, respectively) in triplicate 4 m^3^ enclosures.

	Enclosures	Net calcification rate (μmol·kg^−1^·d^−1^)	POC production rate (μmol·L^−1^·d^−1^)	DOC production rate (μmol·L^−1^·d^−1^)	PIC:POC ratio
HC	1	1.22	12.60	32.30	0.10
	3	1.22	—^a^	31.90	—
	5	1.36	27.80	24.80	0.05
	Mean	1.27	20.20	29.67	0.08
	SD	0.08	10.75	4.22	0.04
LC	2	1.44	3.72	21.00	0.39
	4	1.64	9.15	10.90	0.18
	6	1.65	13.60	10.60	0.12
	Mean	1.58	8.82	14.17	0.23
	SD	0.12	4.95	5.92	0.14

^a^POC samples were not collected in this enclosure during the experiment.

## Discussion

### Enhanced biomass and production under HC treatment

As we mentioned above, acidification in eutrophic waters is not only accompanied by but also coupled to the development of eutrophication^3^. In natural sea water, typically the DOC concentration is less than 100 µM, and a least half of which is resistant to biological degradation^17^. In coastal eutrophic seawater, the concentration of labile DOC is high. In our system, the initial concentration was more than 200 µM. It is obvious that the decomposition of DOC led to the high *p*CO_2_ in the initial water (805 µatm). Although the initial *p*CO_2_ in the water in our study was much higher than the atmospheric CO_2_, the continued aeration in HC enclosures further exacerbated the pre-existing ocean acidification and reduced the pH (800–1000 ppm, Figs 1 and 2). The continued aeration of 400 ppm air reflects the initially high *p*CO_2_ water gradually returning to the air sea equilibrium state (400–800 ppm) by the exchange with ambient *p*CO_2_ air. Our results confirm the two states. The mean *p*CO_2_ levels in the HC and LC treatments were 879.15 ± 145.76 and 658.05 ± 113.98 ppmv, with the significant differences of 221.10 ppmv in the first 4 days (Fig. 2b). Therefore, the results of this study may inform us about phytoplankton responses during a similar natural bloom in current low-pH coastal eutrophic environments under higher atmospheric CO_2_ concentrations in the future.

Many previous mesocosm studies were conducted in high latitude waters (Table S1), where low temperatures lead to high solubility of CO_2_, and potentially larger biological effects of acidification^6, 7^. Our work is the first mesocosm CO_2_ perturbation study in a eutrophic subtropical ecosystem (24.5°N, 118.2°E) and thus could be comparable to the previous mesocosm experiment in the coastal waters of Korea (34.6°N and 128.5°E)^14, 18^. However, the Korean experiments were performed in winter. More importantly, these previous studies were done by adding inorganic nutrients to trigger algae blooms. While, we used natural sea water after filtering out particles (< 0.01 µm). The high nutrient concentration is its own characteristics (eutrophication in Chinese coastal water). Nutrients concentrations, nutrients compositions (N/P/Si, NH_4_^+^/NOx, DIN/DON) and other factors (such as metals) were the same as the *in-situ* seawater with a salinity of 27.3.

In the HC treatment, clear enhancement of phytoplankton total Chl-*a* biomass and diagnostic pigment concentrations of both diatom and prymnesiophytes were observed (Fig. 6). These results are consistent with the production of particulate and dissolved organic carbon in the HC enclosures (Table 1). The increases in pH and DIN/DIC/*p*CO_2_ drawdown due to phytoplankton growth during the experiment were also larger in the HC scenario than those of the LC treatment (Figs 1–3). Similar to our results, increasing inorganic carbon concentrations have been shown to enhance primary production^19^ and carbon assimilation in various photoautotrophs. Of course, studies have reported similar results for diatoms^20^ and coccolithophores^21^ in mono-culture. Growth of a natural phytoplankton community was also stimulated by elevated CO_2_ in an Arctic mesocosm experiment, leading to enhanced nutrient uptake and higher biomass build-up right after dissolved inorganic nutrient addition^16^. The enhanced production and exudation of organic matter in particular stimulated microbial loop activities and altered food web structure^7^.

It has been suggested that CO_2_ could limit carbon fixation by marine phytoplankton and by large diatoms in particular, as the free CO_2_ concentration is usually below that required for half saturation of Ribulose-1, 5-bisphosphate Carboxylase Oxygenase (RUBISCO), the core carbon-fixing enzyme in photosynthesis. There is experimental support for this idea^20, 22^, even though most phytoplankton can utilize cellular C-concentrating mechanisms (CCM) based on the active uptake of CO_2_ and/or HCO_3_^−^ from the environment^23^. The quite high pH values (8.90) and low *p*CO_2_ in water (31 µatm) on day 10 in our HC treatments also indicate that there might be insufficient CO_2_ for phytoplankton (Figs 1 and 2). For diatoms (*Phaeodactylum tricornutum*), previous studies showed that growth and photosynthetic carbon fixation rates were enhanced by the enrichment of CO_2_ under low or moderate levels of light^24^, though photosynthetic inorganic carbon affinity was down regulated by 20% under the high CO_2_ condition^22^. Therefore, it is reasonable to infer that the growth of phytoplankton in eutrophic water, in particular that of large diatoms^25^, can be enhanced by high CO_2_ (Fig. 6 and Table 1).

However, there are also reports of neutral responses and even negative effects^26^. Species-specific CO_2_ responses could result from taxonomic differences among phytoplankton in the physiological mechanisms of CO_2_ uptake^27^. In addition to increased *p*CO_2_ in seawater under HC, lowered pH can lead to acidic stress. The lower calcification rates (Table 1) reflect a more rapid dissolution of CaCO_3_ or reduced rates of calcification in the low pH enclosures^28^. The difference on the ratios of PIC:POC between treatments suggest two opposing effects on the production of organic and inorganic carbon, respectively (Table 1). The lowered pH may also reduce the ability of some species to tolerate high light stress, resulting in increases in respiratory carbon loss^29^. Thus, whether or not phytoplankton will benefit from increased CO_2_ remains controversial^26^, since species-specific behavior and different physiological processes in different waters or experimental conditions are involved^29, 30^. In eutrophic coastal waters where diatoms dominated in the phytoplankton community, our results support the general proposition that increasing CO_2_ may promote phytoplankton photosynthesis and growth^7, 15^.

### Species composition under high CO_2_

Changes in dissolved aqueous CO_2_ may affect phytoplankton community structure^31^. A meta-analysis of published experimental data emphasized that the differing responses to elevated *p*CO_2_ caused sufficient changes between phytoplankton types in competitive fitness to significantly alter community structure^32^. Our results indicate a rapid response of prymnesiophytes in the HC treatment, while a short time-lag was observed in the stimulation of diatom growth (Figs 6 and 7). This response was the opposite of the expected trend, by which large phytoplankton should grow faster than small phytoplankton in nutrient replete conditions while the latter have the advantage in oligotrophic environments. This unexpected result could be due to the greater contribution of NH_4_^+^ to total DIN compared to NO_3_^−^, as prymnesiophytes began to decline in day 6 when NH_4_^+^ was depleted (Figs 3 and 6). Results of an Arctic mesocosm experiment also indicated a positive effect of HC on pico- and nano-eukaryotes during the nutrient replete phase^16^. The authors of this study suggested that if cells are small enough that their dissolved inorganic carbon supply needs can be met at least partly by diffusion, higher seawater CO_2_ concentrations could stimulate photosynthetic carbon fixation and growth in these species^16^.

Our study indicates that diatoms have an overall advantage in eutrophic HC waters compared to prymnesiophytes, although the initial Chl-*a* concentrations of both groups were the same and the initial cell numbers of prymnesiophytes were even higher than those of diatoms (Fig. 6). As mentioned above, the decreased affinity for HCO_3_^−^ or/and CO_2_ and down-regulated CCM in diatom can save CCM-operational energy^22, 33^, so that increased CO_2_ availability can be beneficial in terms of energetics. In contrast, prymnesiophytes could have a competitive advantage over diatoms in low CO_2_ environments^34^. Similarly, it was observed a clear succession from prymnesiophytes to diatoms when CO_2_ concentrations increased from 150 to 750 ppmv^31^. Another experimental study in the North Atlantic Ocean, however, showed a shift away from diatoms and towards coccolithophores under HC, warmer conditions^35^. It has been suggested that paleooceanographic data showing lower Si:N utilization ratios by phytoplankton during the last glacial maximum could be due to community shifts towards non-siliceous species such as prymnesiophytes caused by decreased CO_2_ in the glacial atmosphere (180 ppmv)^31^, although other studies have attributed lower Si:N utilization ratios and export of biogenic Si to relaxed iron limitation of diatoms during glacial periods^36^. In addition, other factors are likely to be also important, such as light. Prymnesiophytes can grow fast and make dense blooms, but they also like high light conditions^37^. In a mesocosm where Chl-*a* reaches such high concentrations, light must be much reduced through self-shading, possibly not favoring prymnesiophyte growth.

### Effects on stoichiometry

Overconsumption of carbon relative to nutrient uptake has been reported in several studies of the planktonic response to increased CO_2_^9, 20, 38^. For the same uptake of inorganic nutrients, net community carbon consumption under increased CO_2_ exceeded present rate by 27 and 39% in 700 and 1050 μatm respectively^1^. However, many other studies have found contrary trends or no effect of high CO_2_ on C:N ratios ^10, 13, 14, 18, 39^. Differing effects of HC on stoichiometric uptake ratios (Table S1) may be attributed to the different biogeochemical demands of the dominant plankton functional types, and life stage-specific biogeochemical requirements^13^.

Our results indicate a clear increase in N:P and N:Si consumption ratios under HC treatments throughout the experiment, supporting higher Chl-*a* and production (Figs 3–6 and Table 1). In other words, assimilations of SRP and SiO_3_^2−^ may be reduced relative to that of nitrogen in eutrophic coastal environments under high CO_2_. A previous study reported that C:P, in contrast to the C:N response, increased significantly in the post-bloom phase^18^. An Arctic mesocosm project with higher CO_2_ had higher POC/POP and PON/POP during the nutrient rich phase^16^. Based on our results, the differences in N:P and N:Si consumption ratios between the two treatments (Figs 4 and 5) were completely due to changes in nitrate drawdown (Fig. 3C). Previous study revealed that elevated *p*CO_2_ strikingly reduced NO_3_^−^ uptake and assimilation in the diatom *Thalassiosira pseudonana* at both high and low light, as indicated by both short-term and steady-state net NO_3_^−^ uptake rates, which was further supported by the reduced gene transcription, protein expression and enzymatic activity of nitrate reductase at high CO_2_^33^. In parallel, diminished NO_3_^−^ uptake at elevated *p*CO_2_ resulted in lower PON and total protein content^40^. This could be more important for larger diatoms as they require a greater fraction (by 3.5-fold compared with small ones) of their total cellular nitrogen to RUBISCO for maintaining carbon fixation, hence a higher nitrogen cost in larger diatoms for RUBISCO leads to higher nitrogen requirements^25^. In addition, accumulating evidence suggests that the nitrogen cycle may respond strongly to higher *p*CO_2_ through increases in global N_2_ fixation^41^ and possibly denitrification^42^, as well as decreases in nitrification^43^.

In contrast, to date, most studies have found negligible or minor effects of projected future changes in *p*CO_2_ on most phytoplankton phosphorus requirements^10^. If indeed N:P and N:Si consumption ratios are elevated under HC environment, the P and Si limitation often observed in fresh and coastal waters will be possibly eased by lower consumption of SRP and Si under HC conditions, relative to the same uptake of DIN (Figs 3 and 4). This is also likely to be the cause of higher Chl-*a* biomass in HC environments with the same initial nutrient concentrations (Fig. 6).

Moreover, the ongoing increase in wind-driven upwelling^44^ and anthropogenic nutrient inputs in coastal systems^45^ may increase nutrient inputs and blooms in coastal waters^46^. The sustained increase in nitrate loading from the Mississippi River^47^, the Pearl River^48^ and the Changjiang River^49^ has resulted in rapidly rising nutrient ratios (N:P and N:Si) since the 1950s, due to increased use of agricultural fertilizers. Associated with the higher Chl-*a* biomass, species succession and eased P and/or Si limitation discussed above, our study further suggests that future climate and land use changes may result in even more serious and complicated interactive effects of eutrophication, ocean acidification and hypoxia in coastal waters^3, 50^.

## Conclusions

Shallow coastal areas are vulnerable to the effects of human development, and can receive massive loads of fresh water, nutrients, and organic and inorganic carbon. In this study, a mesocosm CO_2_ perturbation study was conducted to investigate the effect of rising CO_2_ on a model plankton community in a eutrophic subtropical bay in China. Although the initial DIC and further decomposition of organic matter led to the *p*CO_2_ in the coastal water being much higher than that in the air, further enrichment of CO_2_ appeared to be conducive to the production and biomass of both diatoms and prymnesiophytes. Diatoms had a clear advantage in this highly eutrophic water under the elevated CO_2_ concentration. However, prymnesiophytes seemingly responded rapidly in the HC enclosures, whereas a time lag was observed in diatom growth. Compared with the low CO_2_ treatments, the N/P and N/Si consumption ratios significantly increased during the growth of phytoplankton at higher CO_2_ partial pressure. These results indicate complex effects induced by ocean acidification in phytoplankton stoichiometry, production and community structure in eutrophic coastal waters which may have serious consequences for these biologically and economically important ecosystems.

## Material and Methods

### Experimental setup and sampling

The Xiamen University mesocosm facility for ocean acidification impacts study (FOANIC-XMU, http://mel.xmu.edu.cn/dynamicfile.asp?id=76) was deployed in Wuyuan Bay, Xiamen, China (24.5°N, 118.2°E)^51^. The dimensions of the floating platform are 28 × 10 m, and the facility includes 9 mesocosm enclosures immersed in the seawater along the south side of the platform to avoid shading (Supplementary Material, Fig. S1). The enclosures are 3 m deep and 1.5 m wide, with 50 cm projecting above the sea surface. The volume of the enclosures was 4 m^3^, and they were composed of a 0.9 mm thick cylindrical transparent thermoplastic polyurethane plastic membrane that is partially transparent to UV. The mesocosms are covered with plastic domes to reduce the contamination risk and prevent rainfall from diluting the experiments.

In order to minimize the influences of other groups of organisms such as grazers, and remove non-living suspended particles that would potentially affect the later measurements of biogenic elements, *in situ* seawater was filtered through a water purifier (MU801-4T, Midea) which was equipped with 0.01 μm pore size cartridges, and simultaneously injected into the enclosures. Then 0.2 g L^−1^ of NaCl solution was added into each mesocosm to determine the exact volume by comparison of the salinity before and after salt addition. Ocean acidification conditions were induced gradually with aeration using CO_2_ enrichlors (CE-100, Wuhan Ruihua Instrument & Equipment, China). The *p*CO_2_ of seawater in 6 of the enclosures was perturbed by bubbling with free air (ambient CO_2_, ~400 ppmv, denoted LC) or using an air/CO_2_ mixture at the concentration of 1000 ppmv (denoted HC). The air with different CO_2_ concentrations was delivered into the seawater at a flow rate of ~5 L min^−1^ with 6 mm diameter plastic tubing, and dispersed by an air stone disk placed in the center of each mesocosm’s bottom. The bubbling was continued for the duration of the whole experiment.

The species interactions of natural populations are typically extremely complicated, depending on the abundance and intrinsic properties of various species as well as other abiotic and biotic factors. Therefore, four well-studied phytoplankton species were used in this study, including the diatoms *Phaeodactylum tricornutum* (CCMA 106, from Center for Collections of Marine Algae, Xiamen University) and *Thalassiosira weissflogii* (CCMP 102, from The National Center for Marine Algae and Micobiota, USA), and the coccolithophores *Emiliania huxleyi* (CS-369, from Commonwealth Scientific and Industrial Research Organization, Australia) and *Gephyrocapsa oceanica* (NIES-1318, National Institute for Environmental Studies, Japan), were first mono-cultured indoors at 400 and 1000 ppmv CO_2_. They were acclimated for 10 days (about 10–15 generations) at 20 °C and 150 μmol m^−2^ s^−1^ (cool white fluorescence) irradiance using the same eutrophic bay seawater later used for the experiments, collected and filtered *in situ* without nutrient additions. After the acclimation period, these species were inoculated into each enclosure with equivalent chlorophyll *a* (Chl-*a*), respectively, at a total final abundance of 5.07*10^4^ cells L^−1^. The initial abundances of *Phaeodactylum tricornutum*, *Thalassiosira weissflogii*, *Emiliania huxleyi* and *Gephyrocapsa oceanica* were about 10000, 700, 20000 and 20000 cells L^−1^, respectively. The initial cell concentrations were set up based on their differences in size and Chl-*a* per cell to yield similar initial biomass levels. The growth of these phytoplankton groups was studied for 15 days (15 to 30 June, 2013) under present and elevated partial pressure of CO_2_ (*p*CO_2_ = 400 and 1000 ppmv, respectively) in triplicate 4 m^3^ enclosures.

### Environmental factors

The salinity, temperature and pH profiles in the mesocosm were measured daily at 10:00 AM with a CTD (RBR) or a SeaFET (Satalantic). Due to aeration, these profiles indicated the water in each enclosure was homogenized (Supplementary Material, Fig. S2). The subsamples for chemical and biological determinations were taken at the middle of the mesocosms with a water sampler. The total amount of water for sampling from the enclosures was less than 5% of the initial volume. The pH values were determined using the pH indicator meta-cresol purple with a spectrophotometer (Agilent 8453), with a measurement accuracy of ± 0.0005. The CO_2_ partial pressure in the seawater (*p*CO_2_) was calculated by the program CO2SYS^52^ from dissolved inorganic carbon (DIC) and total alkalinity (TA)^53, 54^.

Nutrient samples for NO_3_^−^ + NO_2_^−^ (NO_x_), NH_4_^+^ and soluble reactive phosphate (SRP) were filtered immediately after collection through 47 mm GF/F filters and were then frozen at −20 °C. Samples for SiO_3_^2−^ were filtered through acid-cleaned 0.45 µm pore size acetate cellulose filters and were kept refrigerated at 4 °C. Filtrates for NH_4_^+^ and SiO_3_^2−^ were preserved with 100 μL CCl_4_. All the nutrient samples were measured in our land-based laboratory at Xiamen University within 15 days after the mesocosm experiment. Nutrient samples for NO_X_, SRP and SiO_3_^2−^ were analysed using a four-channel continuous-flow Technicon AA3 Auto-Analyzer (Bran-Lube GmbH)^55^. NO_x_ was analyzed using the copper-cadmium column reduction method^5^. SRP and SiO_3_^2−^ were measured using typical spectrophotometric methods^56^. NH_4_^+^ was run with a 722 type spectrophotometer (Xiamen Analytical Instrument Co., China) according to the indophenol blue spectrophotometric method^5, 57^. The precision for nutrient analyses in this study was ≤3%, and the detection limits for NO_x_, SRP, SiO_3_^2−^ and NH_4_^+^ analyses were 0.03, 0.03, 0.05 and 0.16 μM, respectively.

### Phytoplankton biomass and community structure

Photosynthetic pigment concentrations were measured by high-performance liquid chromatography (HPLC) using a Shimadzu 20 A HPLC system fitted with a 3.5 μm Eclipse XDB C_8_ column (4.6 × 150 mm, Agilent Technologies, Waldbronn, Germany), as in our previous studies^8, 58^. Briefly, seawater samples for phytoplankton pigment analysis (0.2–2 L, according to biomass) were filtered through 25 mm GF/F glass fiber filters (under a vacuum pressure < 10 kPa under dim light), and then they were immediately frozen (−80 °C) until analysis in the laboratory within 30 days. Phytoplankton pigments were extracted in N, N-dimethylformamide and analyzed. The concentrations of chlorophyll *a* (Chl-*a*), diagnostic pigments of diatoms, (fucoxanthin, Fuco) and prymnesiophytes (19′hexanoyl-oxy-fucoxanthin, 19-Hex) were detected, and quantified using standards that were purchased from DHI Water & Environment, Hørsholm, Denmark.

Pigment samples of the four phytoplankton species were collected in exponential growth phase during mono-culture under indoor conditions to obtain the ratios of diagnostic pigments to Chl-*a* for CHEMTAX analyses^8, 58^. These initial pigments ratios might be quite different from those of the same species grown under sunlight in the mesocosms. To confirm the phytoplankton community structure based on pigments information, a 0.5 L water sample was collected and preserved with Lugol’s iodine solution for microscopic observation. The Chl-*a* concentrations were also compared to the cell number, and these two values were significantly correlated (Supplementary Material, Fig. S3, R^2^ = 0.76, n = 27, *p* < 0.01).

### Carbon metabolism

Net calcification rate was estimated by the TA anomaly method^59^. TA was measured by potentiometric titration using the Gran procedure^60^. In addition, TA has to be corrected for the effect of primary production, i.e., since an uptake of 1 mol NO_3_^−^ or PO_4_^3−^ increases TA by 1 mole^59^, and an uptake of 1 mol NH_4_^+^ decreases TA by 1 mole^61^. Thus, the net calcification rate can be estimated as equation (1).

1 $${\rm{Net}}\,{\rm{calcification}}\,{\rm{rate}}=-0.5\times ({\rm{\Delta }}T{A}_{measured}-{\rm{\Delta }}N{{O}_{3}}^{-}-{\rm{\Delta }}P{{O}_{4}}^{3-}+{\rm{\Delta }}N{{H}_{4}}^{+})/{\rm{\Delta }}t$$

For particulate organic carbon (POC) sampling, seawater was filtered through a pre-combusted 47 mm GF/F filter (Whatman). Prior to analysis, the filters were frozen at −20 °C and POC (via acid fuming) was determined with a PE-2400 SERIES IICHNS/O analyzer according to the JGOFS protocols^56^. Dissolved organic carbon (DOC) samples were filtering onto pre-combusted 25 mm GF/F filters and stored in pre-combusted EPA vials at −20 °C until analysis using a Shimadzu TOC-V Analyzer^62^. The overall POC and DOC production rates for 15 days (day 0–day 14) were calculated according to the following equations:

2 $${\rm{POC}}\,{\rm{production}}\,{\rm{rate}}={\rm{\Delta }}POC/{\rm{\Delta }}t$$

3 $${\rm{DOC}}\,{\rm{production}}\,{\rm{rate}}={\rm{\Delta }}DOC/{\rm{\Delta }}t$$

Since particulate inorganic carbon (PIC) samples were limited, we assumed that all PIC is in the form of CaCO_3_. Accordingly, the PIC value in this study was calculated with the following formula^63^:

4 $${{\rm{\Delta }}\mathrm{PIC}}_{calculated}=CaC{O}_{3}\,accumulation=-0.5\times ({\rm{\Delta }}T{A}_{measured}-{\rm{\Delta }}N{{O}_{3}}^{-}-{\rm{\Delta }}P{{O}_{4}}^{3-}+{\rm{\Delta }}N{{H}_{4}}^{+})$$

In all the formulas above, ΔTA, ΔNO_3_^−^, ΔPO_4_^3−^, ΔNH_4_^+^, ΔDOC, ΔPOC, ΔPIC_calculated_ denote the changes of these parameters, and Δt denotes the elapsed time. A one-way ANOVA was used for statistical analysis following a test for homogeneity of the variances. The significance level was set at *p* < 0.05. ANOVA results were compared using the Tukey HSD method.

## Electronic supplementary material

Supplementary Material
