# Supplementary material for: Effects of elevated CO2 on phytoplankton during a mesocosm experiment in the southern eutrophicated coastal water of China
Source: Sci Rep. 2017 Jul 31;7:6868. doi: 10.1038/s41598-017-07195-8 (PMC5537254; doi:10.1038/s41598-017-07195-8)
Supplement: Supplementary file 1 — Supplementary Material [file 41598_2017_7195_MOESM1_ESM.doc]

**Supplementary Material for:**

**Effects of elevated CO_2_ on phytoplankton during a mesocosm experiment in the southern eutrophicated coastal water of China**

Xin Liu^1†^, Yan Li^1†^, Yaping Wu^1†^, Bangqin Huang^1^*, Minhan Dai^1^*, Feixue Fu^2^, David A. Hutchins^2^, Kunshan Gao^1^*

[1] State Key Laboratory of Marine Environmental Science, Xiamen University, 361005 Xiamen, Fujian, China

[2] Department of Biological Sciences, University of Southern California, 3616 Trousdale Parkway, Los Angeles, California, 90089, USA

[†] These authors contributed equally to this work.

[*] Correspondence to: Bangqin Huang (bqhuang@xmu.edu.cn), Minhan Dai (mdai@xmu.edu.cn), Kunshan Gao (ksgao@xmu.edu.cn)

Figure S1.

The mesocosm facility for ocean acidification studies at Xiamen University (FOANIC-XMU, http://mel.xmu.edu.cn/dynamicfile.asp?id=76) in Wuyuan Bay, Xiamen, China (N24º31´48″, E118º10´47″). The photo was taken by Prof. Gao.


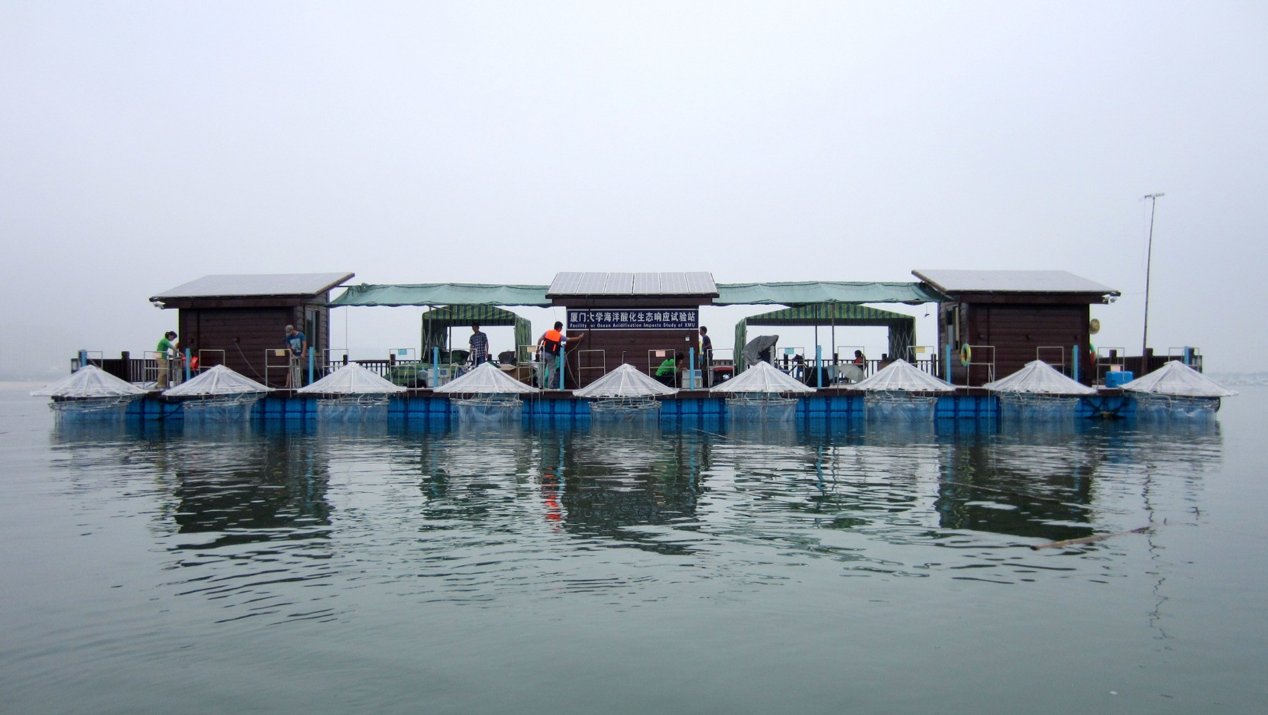


Figure S2.

Vertical profiles of temperature (°C) and salinity in 6 seawater enclosures which were perturbed by bubbling with ambient air (CO_2_, 400 ppmv CO_2_, LC) or an air/CO_2_ mixture at a target concentration of 1000 ppmv CO_2_ (HC) on Day 5 during the mesocosm experiments.

Figure S3

Comparison of the results between the total chlorophyll *a* concentrations and cells abundances.

Table S1

Previous mesocosm experiments on ocean acidification effects: setups and key findings

|  | Engel,  et al.  (2005) | Kim,  et al.  (2006) | Riebesell,  et al.  (2007) | Kim,  et al.  (2011) | Silyakova,  et al.  (2013) | Zark,  et al.  (2015) | This  study |
| --- | --- | --- | --- | --- | --- | --- | --- |
| Time | May-June,  2001 | Nov.-Dec., 2004 | May-June,  2005 | Nov.–Dec., 2008 | May-June, 2010 | March-June 2013 | June,  2013 |
| Location | Bergen,  Norway | Korea | Bergen,  Norway | Korea | Svalbard,  Norway | Kristineberg Sweden | Xiamen,  China |
|  | 60.4 °N,  5.3 °E | 34.6 °N, 128.5 °W | 60.3 °N,  5.2 °E | 34.6 °N, 128.5 °W | 78.9 °N, 11.9 °E | 58.3°N,  11.5°E | 24.5 °N, 118.2 °E |
| System  Volume | 11 m^3^ | 0.15 m^3^ | 27 m^3^ | 3 m^3^ | 50 m^3^ | 50 m^3^ | 4 m^3^ |
| Dominated  Group | Prymne-  siophytes | Diatoms | Diatoms  Prymne-  siophytes | Diatoms | Diatoms  Dinoflage-  llates | NA | Diatoms  Prymne-  siophytes |
| CO_2_ treatments | 410/710 ppmv | 250/400/750 ppmv | 350/700/ 1050 ppmv | 400/900 ppmv | Ranging from 185 to 1420 µatm | 400/900 µatm | 400/1000 ppmv |
| Initial Nutrients | 15.3 µM-N,  0.5 µM-P | 23 µM-N, 0.9 µM-P | 14 µM-N,  0.7 µM-P | 23 µM-N, 0.9 µM-P | 5 µM-N,  0.3 µM-P | NA | 83.8 µM-N,  3.4 µM-P |
| C/ N /P | **+** | **-** | **+** | **-** | **-** | **+** | **+** |
| Biomass/Growth | **+** | **+** | **+** | **+** | **+** | **-** | **+** |

+: phytoplankton stoichiometry (POC/PON/POP, ∆DIC/∆DIN/∆SRP etc.) or growth (Chl-*a*, C biomass, growth rates, primary production, etc.) sensitive to the elevated CO_2_ in nutrient-replete period; -: no clear effect detected; NA: data not available

**References：**

Engel, A. et al. Testing the direct effect of CO_2_ concentration on a bloom of the coccolithophorid Emiliania huxleyi in mesocosm experiments. Limnol. Oceanogr. 50, 493-507, doi:10.4319/lo.2005.50.2.0493 (2005).

Kim, J. M. et al. The effect of seawater CO_2_ concentration on growth of a natural phytoplankton assemblage in a controlled mesocosm experiment. Limnol. Oceanogr. 51, 1629-1636 (2006).

Kim, J. M. et al. Shifts in biogenic carbon flow from particulate to dissolved forms under high carbon dioxide and warm ocean conditions. Geophys. Res. Lett. 38, L08612, doi:10.1029/2011gl047346 (2011).

Riebesell, U. et al. Enhanced biological carbon consumption in a high CO_2_ ocean. Nature 450, 545-548, doi:10.1038/nature06267 (2007).

Silyakova, A. et al. Pelagic community production and carbon-nutrient stoichiometry under variable ocean acidification in an Arctic fjord. Biogeosciences 10, 4847-4859, doi:10.5194/bg-10-4847-2013 (2013).

Zark, M., Riebesell, U. & Dittmar, T. Effects of ocean acidification on marine dissolved organic matter are not detectable over the succession of phytoplankton blooms. Science Advances 1, e1500531, doi:10.1126/sciadv.1500531 (2015).
